# Metal–Organic Framework-Derived Co_9_S_8_ Nanowall Array Embellished Polypropylene Separator for Dendrite-Free Lithium Metal Anodes

**DOI:** 10.3390/polym16131924

**Published:** 2024-07-05

**Authors:** Deshi Feng, Ruiling Zheng, Li Qiao, Shiteng Li, Fengzhao Xu, Chuangen Ye, Jing Zhang, Yong Li

**Affiliations:** 1 Advanced Materials Institute, School of Materials Science and Technology, Qilu University of Technology (Shandong Academy of Sciences), Jinan 250014, China; 2 Department of Digital Media and Animation, Shandong Communication & Media College, Jinan 250200, China; 3 SVOLT Energy Technology Co., Ltd., Changzhou 213299, China; 4 College of Materials and Chemical Engineering, Heilongjiang Institute of Technology, Harbin 150006, China

**Keywords:** lithium metal batteries, polypropylene separator, MOF, transition metal sulfides

## Abstract

Developing a reasonable design of a lithiophilic artificial solid electrolyte interphase (SEI) to induce the uniform deposition of Li^+^ ions and improve the Coulombic efficiency and energy density of batteries is a key task for the development of high-performance lithium metal anodes. Herein, a high-performance separator for lithium metal anodes was designed by the in situ growth of a metal–organic framework (MOF)-derived transition metal sulfide array as an artificial SEI on polypropylene separators (denoted as Co_9_S_8_-PP). The high ionic conductivity and excellent morphology provided a convenient transport path and fast charge transfer kinetics for lithium ions. The experimental data illustrate that, compared with commercial polypropylene separators, the Li//Cu half-cell with a Co_9_S_8_-PP separator can be cycled stably for 2000 h at 1 mA cm^−2^ and 1 mAh cm^−2^. Meanwhile, a Li//LiFePO_4_ full cell with a Co_9_S_8_-PP separator exhibits ultra-long cycle stability at 0.2 C with an initial capacity of 148 mAh g^−1^ and maintains 74% capacity after 1000 cycles. This work provides some new strategies for using transition metal sulfides to induce the uniform deposition of lithium ions to create high-performance lithium metal batteries.

## 1. Introduction

Lithium-ion batteries (LIBs), with their high energy density and mature preparation technology, have been fully integrated into human daily life. However, with the rapid development of new technologies, especially in the field of electric vehicles and grid-scale energy storage systems, there is an urgent need to further enhance the energy density and cycle stability of LIBs [1,2,3]. Lithium metal has an ultra-high theoretical capacity (3860 mAh cm^−2^) and a lower electrochemical potential (−3.04 vs. a standard hydrogen electrode) and is considered the “holy grail” anode material for next-generation energy storage materials [4,5]. Unfortunately, the inherent problems of lithium metal anodes (LMAs) seriously affect their practical application. For example, the high reactivity of Li metal is known to cause uneven dendritic growth during the plating/stripping process, raising concerns about safety and battery life [6]. In addition, the unstable solid–electrolyte interphase (SEI) layer formed on the surface of LMAs cannot inhibit the growth of lithium dendrites [7]. These lithium dendrites will again react with the electrolyte after puncturing the SEI layer. This repeated process will continuously consume active Li and the electrolytes of the batteries. At the same time, the larger dendrites will break off and form “dead Li”. During this continuous reaction process, the SEI is damaged and will regenerate [8]. As a result, the SEI film will become thicker and more inhomogeneous, which will accelerate the inhomogeneous Li plating. This process leads to reductions in the Coulombic efficiency (CE) and rapid capacity fade and also poses a significant safety hazard [9,10].

Massive efforts have been devoted to finding ways to improve the stability of LMAs. Some strategies have been proven to be expected to make a positive and effective contribution to suppressing Li dendrites, including designing 3D porous skeleton collectors [11,12,13,14], tailoring the physical and chemical properties of SEI using various electrolyte additives or solvents [15,16,17,18,19], and applying an artificial SEI consisting of inorganic materials [20,21,22], polymers [23,24,25,26], or their hybrids [27,28,29] on the surface of LMAs. However, all of these strategies have their limitations. The higher specific surface area of the 3D porous skeleton collector increases the contact between LMAs and the electrolyte, which often leads to serious side reactions [30]. The electrolyte solvents and additives are often expensive and will be depleted with cycling [31]. Although designing an artificial SEI can effectively improve the stability of LMAs, the process has very harsh environmental requirements due to the high activity of lithium metal, and it is difficult to use widely in industrialization [32]. Coating the surface of the separators rather than the LMAs with a protective layer will effectively avoid the shortcomings of the above strategies [33,34,35]. For example, Cui et al. reported a strategy to form a uniform Li_2_S protective layer to suppress dendrite growth [33]. The uniform and high ionic conductivity Li_2_S coating enhanced the protective function of the layered SEI. Song et al. decorated lithiophilic magnesium (Mg) nanoparticles on the surfaces of the separators to reduce the Gibbs free energy of Li electrodeposition. Mg nanoparticles serve as heterogeneous nucleation sites, which can guide uniform Li plating [36]. In addition, various materials have been developed to improve the properties of separators to enhance the stability of LMAs, including inorganic materials (Al_2_O_3_ [37,38], SnS_2_ [39], graphene [40,41], and zeolite [42]), MOF [43,44,45,46,47], COF [48], etc. The question of how to find an ideal material with superior Li^+^ ion conductivity, high lithiophilicity, and industrialization prospect still needs further exploration [39,49].

Herein, we report a facile approach to prepare well-aligned Co_9_S_8_ nanowall arrays on the surface of the polypropylene separator (Co_9_S_8_-PP) by vulcanizing the in situ growth of an MOF array on PP separators (MOF-PP). The well-designed multifunctional separator could effectively homogenize the Li^+^ flux, suppressing dendrite growth, and improve the electrochemical performance of LIBs. The Co_9_S_8_ nanowall array can act as a barrier to suppress dendrite growth in high-performance LIBs without any significant increase in the weight or a change in the volume. Owing to the lithiophilicity of Co_9_S_8_, this well-aligned nanowall array can make Li^+^ uniformly and rapidly migrate. Due to the polarity and conductivity of Co_9_S_8_, LMB with a Co_9_S_8_-PP separator realizes dendrite-free generation under long cycling. The Co_9_S_8_-PP separator provides excellent cycle stability and cycle-specific capacity for lithium metal full cells and performs 800 cycles at a rate of 1 C. This shows that the Co_9_S_8_-PP separator has broad prospects in practical applications.

## 2. Experimental Section

Preparation of MOF-PP: First, 5.2 g of 2-methylimidazole and 2.34 g of Co(NO_3_)_2_·6H_2_O were added to 150 mL of deionized water and stirred until completely dissolved. Subsequently, a polypropylene (PP) separator was placed in the mixed solution and allowed to float in the solution for 10 h. Finally, the PP separator was removed, cleaned, and vacuum-dried. During this process, the MOF material was grown on the side of the PP separator soaked in the solution. The growth mass of MOF on the PP separator was about 0.37 mg cm^−2^.

Synthesis of Co_9_S_8_-PP: An amount of 1.2 g thioacetamide (TAA) was dissolved in 80 mL deionized water and transferred to an autoclave. Subsequently, the fabricated MOF-PP separator was soaked in the above solution and reacted at 100 °C for 6 h. Finally, the samples were removed, cleaned, and dried under a vacuum, and Co_9_S_8_-PP material was obtained. The mass load of Co_9_S_8_ on the PP separator was about 0.16 mg cm^−2^.

Material characterization: The as-obtained separators were initially characterized by X-ray diffraction (XRD, Rigaku D/Max-r B, Cu-Kα). The morphological observation was conducted on a field emission scanning electron microscope (FESEM, SM-74190UEC JEOL, Japan Electronics Co., Ltd) at 15 KV and a high-resolution transmission electron microscope (HRTEM, JEM 2100F).

Electrochemical measurements: A series of standard CR2016 coin cells were assembled to test the electrochemical behavior of different separators. A 1 M solution of LiPF_6_ mixed in dimethyl carbonate (DMC) and ethylene carbonate (EC) with a volume ratio of 1:1 was used as the electrolyte, and commercial PP film (Celgard 2400, USA) was used as the separator. The amount of electrolyte used was about 1.78 μL mAh^−1^. LiFePO_4_ (LFP) cathode sheet was prepared by mixing LFP powder with polyvinylidene fluoride and carbon black in the weight ratio of 8:1:1; N-methyl pyrrolidone was used as the solvent and then pasted on aluminum foil. The active material density of the cathode was about 11.0 mg cm^−2^. The prepared LFP cathodes were paired with bare Li metal anodes to form a full cell for electrochemical testing, with PP, MOF-PP, and Co_9_S_8_-PP as the separators. All of the symmetric and full cells were assembled in a glove box filled with argon atmosphere (O_2_ < 0.1 ppm, H_2_O < 0.1 ppm). The electrochemical impedance spectroscopy (EIS) was performed on an electrochemical workstation (CHI760E) in the frequency range of 100 kHz ~1 Hz. The Coulombic efficiency and cyclic stability of the cells were measured with a battery test system (LAND CT2001A).

## 3. Results and Discussion

**Figure 1:**
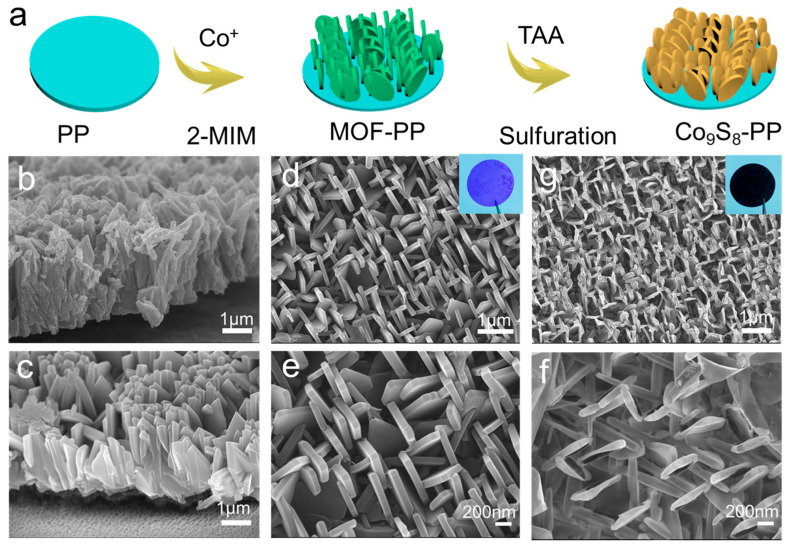
(**a**) Design strategy for Co_9_S_8_-PP separator. (**b**,**c**) SEM image of cross-sectional morphologies of Co9S8-PP and MOF-PP. (**d**,**e**) Top-surface morphologies of MOF-PP at various magnifications and optical picture in upper right corner. (**g**,**f**) Top-surface morphologies of Co9S8-PP at various magnifications and optical picture in upper right corner.

Figure S1 shows the optical photo and SEM images of the commercial PP separator, which has many tiny pores suitable for Li^+^ transmission. The fabrication procedure of the dendrite-free Co_9_S_8_ nanosheet arrays on polypropylene separators (Co_9_S_8_-PP) is illustrated in Figure 1a. Firstly, well-arranged MOF nanosheet arrays were grown on the PP separator surface by a simple in situ growth method, immersing the polypropylene separator (PP) in the precursor solution containing 2-methylimidazole and Co(NO_3_)_2_·6H_2_O for several hours. Figure S2 (Supporting Information) shows the optical photos of the prepared MOF-PP separator, and the simple synthesis scheme allows for the large-scale manufacturing of MOF-PP separators. It is worth noting that the MOF array was only grown on one side of the PP separator that faces the lithium metal anode, and the other side is exposed. This means that the Co_9_S_8_-modified separator is similar to an ordinary separator and will not cause batteries to short-circuit. The picture of the folded MOF-PP in Figure S2c shows its good mechanical properties. In addition, the corresponding element mapping image proves that the MOF array grew uniformly on the PP separator (Figure S3). 

Subsequently, the prepared MOF-PP was converted to Co_9_S_8_-PP by chemical solvothermal sulfurization, and its color was also completely transformed from the blue of the MOF array (inset of Figure 1d) to the black of Co_9_S_8_ (inset of Figure 1g), which means that Co-MOF is completely converted to Co_9_S_8_. Figure S4 shows the XRD pattern of the prepared Co_9_S_8_-PP separator. Some characteristic peaks in the XRD pattern of Co_9_S_8_-PP correspond to those of Co_9_S_8_ (JCPDS No. 19-0364), which demonstrates that Co-MOF can be easily converted to Co_9_S_8_ by solvothermal sulfurization at low temperatures (100 °C). The corresponding element mapping and atomic mass fraction image further prove the successful conversion of MOF to Co_9_S_8_ (Figure S5). Since cobalt and sulfur have different diffusion rates, the Kirkendall effect between them forms a Co_9_S_8_ array with uniform vertical growth and distribution. Figure S6 displays the excellent mechanical properties of the obtained Co_9_S_8_-PP and confirms that it can be used directly as a separator for lithium metal batteries. The cross-sectional scanning electron microscope (SEM) images of Co_9_S_8_-PP and MOF-PP are shown in Figure 1b,c. We can see that the MOF array (Figure 1c) is flat and arranged vertically on the PP separator. Even after solvothermal sulfurization, the obtained Co_9_S_8_ array still maintains the original morphology. Compared with MOF-PP, the thickness of the Co_9_S_8_-PP separator obtained by solvothermal sulfurization did not change (Figure S7). This neatly arranged lithiophilic Co_9_S_8_ array can ensure the uniform transmission of Li^+^ ions and suppress the formation of Li dendrites. Figure 1d–g show the top-surface morphologies of the MOF-PP and Co_9_S_8_-PP separators. The morphologies of the Co_9_S_8_ array obtained by solvothermal sulfurization have no obvious change, and the neatly arranged nanoarray lays a good foundation for the construction of a uniform Li^+^ channel. From the above synthesis process to morphology characterization, it can be concluded that we synthesized a simple, low-cost, and scalable strategy for the in situ growth of a lithium metal battery multifunctional defender on PP separators.

**Figure 2:**
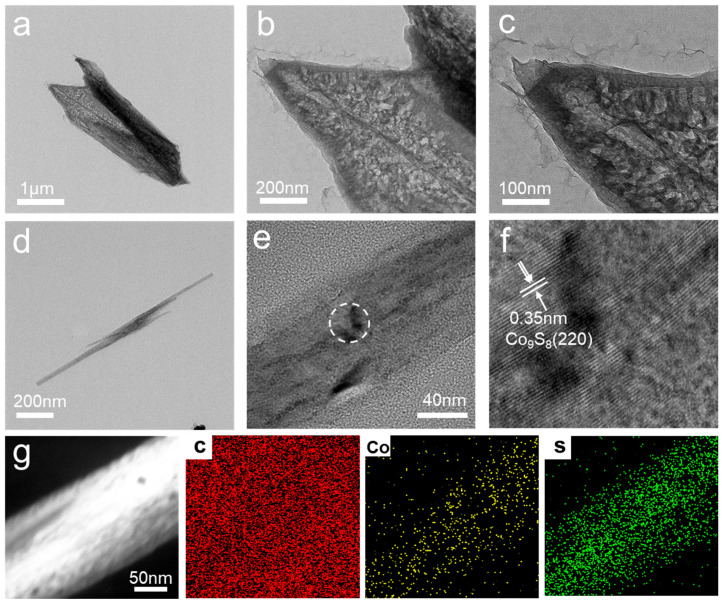
(**a**–**c**) Low-resolution TEM image of MOF-PP. (**d**) Low-resolution TEM image of Co_9_S_8_-PP. (**e**,**f**) High-resolution TEM image of Co_9_S_8_-PP. (**g**) EDX mappings of Co_9_S_8_-PP.

To further observe the microstructure of the synthesized material, we performed transmission electron microscope (TEM) characterization of the MOF and Co_9_S_8_ arrays. Figure 2a shows the low-resolution TEM image of the MOF array grown in situ on the PP separator in which the shape of the MOF is flat and the middle is hollow. We can see that there are many MOF crystals on the surface via high-resolution TEM (Figure 2b,c), indicating that the MOF is successfully grown in situ on the PP separator as a nanosheet structure after synthesis. Figure 2d shows part of the morphology of Co_9_S_8_. Incomplete peeling resulted in a broken portion of Co_9_S_8_. The magnified TEM image shows that Co_9_S_8_ was successfully converted (Figure 2e). The high-resolution TEM image shows a labeled lattice spacing 0.35 nm, corresponding to the (220) plane of Co_9_S_8_ (Figure 2f). Figure 2g shows the energy-dispersive X-ray spectroscopy (EDX) elemental mapping of Co_9_S_8_. Cobalt and sulfur are uniformly dispersed on the Co_9_S_8_ wall, which means that MOF is successfully converted to Co_9_S_8_ after solvothermal sulfurization.

XPS spectroscopy briefly analyzes the valence states of elements (Figure S8). As shown in Figure S8, due to Co 2p^3/2^ and Co 2p^1/2^ in the cobalt–sulfur compound, it shows strong binding energy at 778.5 eV and 793.6 eV. Owing to Co_9_S_8_-PP exposure in air, the shoulder peaks at 780.5 eV and 797.3 eV are related to Co 2p^3/2^ and Co 2p^1/2^ from Co-O. The weak binding energies at 784.2 eV and 802.2 eV can be determined as the shake-up peaks of Co^2+^. The results of XPS and TEM both indicate the formation of Co_9_S_8_.

**Figure 3:**
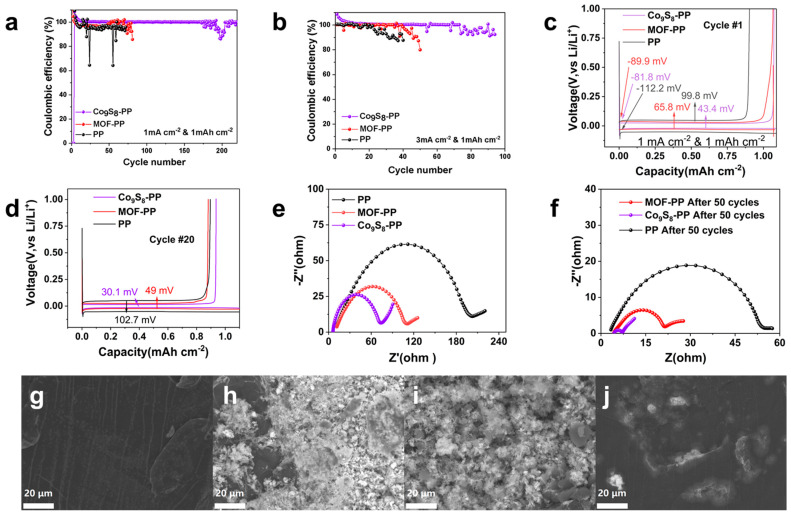
The electrochemical performance of the Li//Cu half-cells with different separators: (**a**,**b**) the Coulombic efficiency of Li plating/stripping at (**a**) 1 mA cm^-2^ and 1 mAh cm^−2^ and at (**b**) 3 mA cm^-2^ and 1 mAh cm^-2^. The voltage profiles at (**c**) the 1st cycle and (**d**) 20th cycle for the cells with a fixed Li deposition amount of 1 mAh cm^-2^. EIS curves after (**e**) 1 and (**f**) 50 cycles at 1 mA cm^-2^ and 1 mAh cm^-2^. SEM images of (**g**) fresh Li foil, cycled Li foil in the control cell with the (**h**) PP separator, (**i**) MOF-PP separator, and (**j**) Co_9_S_8_-PP separator after 200 cycles.

To explore the effect of the prepared Co_9_S_8_-PP separator on the cycle performance of LMAs during plating/stripping, Li//Cu asymmetrical cells were assembled with PP, PP-MOF, and PP-Co_9_S_8_ as separators, respectively. As shown in Figure 3a, the half-cell with an ordinary PP separator showed poor reversibility and short-circuited after 70 cycles at a current density of 1 mA cm^−2^ and a capacity of 1 mAh cm^−2^. This is mainly because commercial PP separators cannot guarantee uniform Li stripping, and a large number of lithium dendrites are formed on the surface of the lithium anode. Numerous Li dendrites pierce the PP separator and easily result in a short circuit. Although the CE of the half-cell with the MOF-PP separator was improved, it still decays quickly. However, the performance of the Li-Cu cell with the Co_9_S_8_-PP separator has been greatly improved, and the CE is still higher than 99% even after 180 cycles. This should be attributed to the high lipophilic Co_9_S_8_ improving the Li^+^ flux, inhibiting the formation of dendrites, and reducing the generation of “dead lithium”, thus improving the utilization rate of active lithium and enhancing the CE. The same trend occurs when the current density increases to 3 mA cm^−2^ (Figure 3b). The CE of cells with a Co_9_S_8_-PP separator is greatly improved. After 70 cycles, the CE retention rate is 98%. This shows that the Co_9_S_8_-PP separator can also suppress the formation of dendrites and avoid the generation of “dead Li” even at high current densities. This fully demonstrates that the plating/stripping behavior of Li metal can be regulated via the Co_9_S_8_ separator. 

To further explore the mechanism by which the Co_9_S_8_ separator improves the reversibility of LMAs, the voltage distributions of Li//Cu half-cells with three different separators are compared (Figure 3c,d). The voltage drops of Li//PP//Cu, Li//PP-MOF//Cu, and Li//PP-Co_9_S_8_//Cu half-cells during the initial lithium plating cycle were 112.2 mV, 89.9 mV, and 81.8 mV, respectively, which can be used to evaluate the nucleation overpotential of lithium metal. This is mainly related to the Co^+^ lithiation process of the nanosheet array and the formation of S-enriched SEI. The solvothermal sulfurization Co_9_S_8_ array can observably reduce the overpotential of Li nucleation. Moreover, the voltage hysteresis of the cell with a PP-Co_9_S_8_ separator (43.4 mV) was smaller than that of the cells with PP-MOF (65.8 mV) and a PP separator (99.8 mV), indicating that the charge transfer resistance is greatly reduced. As shown in Figure 3d, the voltage hysteresis of the Li//PP//Cu cell increased to 102.7 mV after 20 cycles, which was mainly caused by the accumulation of unstable SEI. In contrast, the voltage hysteresis of the cells using the MOF-PP separator (from 65.8 mV to 49 mV) and the CO_9_S_8_-PP separator (from 43.4 mV to 30.1 mV) after 20 cycles did not rise but fell, proving that the presence of sulfur is conducive to the formation of a sturdy and stable SEI film. The absorption efficiency of different separators for electrolytes was tested (Figure S9). The results show that the electrolyte absorption rate of the Co_9_S_8_-PP separator was 95% higher than that of the pure PP separator. Figure 3e,f show the Nyquist curves of the half -cells equipped with different separators. The high-frequency semicircles and low-frequency sloping lines in the Nyquist curves correspond to the charge transfer resistance (R_ct_) and the Li^+^ diffusion resistance within the electrodes, respectively [50,51]. Compared with the cells with PP-MOF and PP separators, Li//PP-Co_9_S_8_//Cu has a significantly lower R_ct_ and a better electrochemical environment. Thanks to the excellent electrolyte absorption and improved wettability of the CO_9_S_8_-PP separator, the R_ct_ of the Li//Co_9_S_8_//Cu half-cell becomes smaller after 50 cycles. In addition, the low-frequency sloping lines prove that Li//PP-Co_9_S_8_//Cu has lower Li^+^ diffusion resistance. 

To further explore the influence of different separators on the cycling process and verify our inference, the surface morphology of Li anodes after 200 cycles (1 mA cm^−2^, 1 mAh cm^−2^) was observed by SEM. Compared with the uncycled Li metal anode (Figure 3g), a large number of lithium dendrites were distributed on the surface of the lithium anode with a PP separator, accompanied by a significant accumulation of “dead lithium” (Figure 3h). This is the main reason for the reduction in the CE, and a large amount of dendrites may also puncture the separator and cause battery failure. The Li anodes using an MOF-PP separator have reduced Li dendrites after 200 cycles, but they are unable to completely suppress dendrite growth (Figure 3i). Notably, the lithium anode using the Co_9_S_8_-PP separator showed no significant dendrite formation on the surface after 200 cycles (Figure 3j). This fully demonstrates that the Co_9_S_8_ array can effectively homogenize Li^+^ ion flux, enhance the SEI, and inhibit the growth of lithium dendrites.

**Figure 4:**
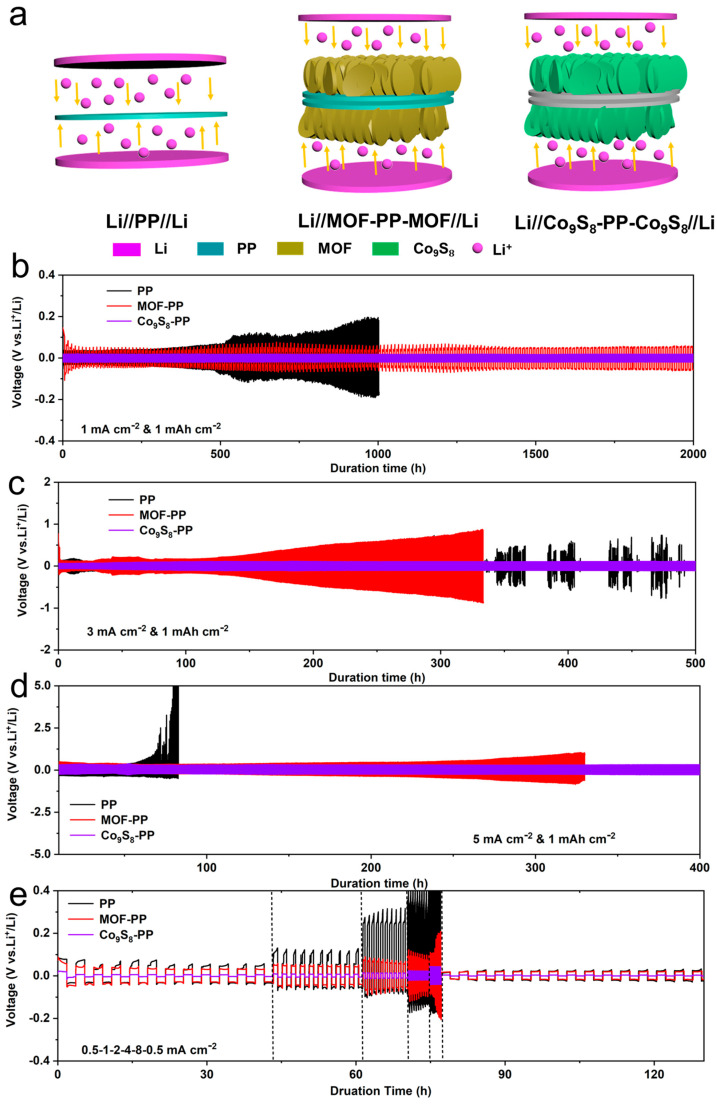
Electrochemical behavior of three different symmetric batteries. (**a**) Schematic diagram of symmetric cells assembled with different separators. Symmetric cell long-term cycling of Li//PP//Li, Li//MOF-PP-MOF//Li, and Li//Co_9_S_8_-PP-Co_9_S_8_//Li with current densities and Li plating/stripping capacities of (**b**)1 mAh cm^−2^ and 1 mA cm^−2^; (**c**) 1 mAh cm^−2^ and 3 mA cm^-2^; and (**d**) 1 mAh cm^−2^ and 5 mA cm^−2^. (**e**) Rate characterization of symmetric batteries at current densities of 0.5, 1, 2, 4, and 8 mA cm^−2^ with capacity of 1 mAh cm^−2^.

A series of symmetric batteries was assembled with different separators under a fixed capacity of 1 mAh cm^−2^, and their galvanostatic cycling stability was compared. Figure 4a shows a schematic diagram of the assembled symmetric cells with different separators. As shown in Figure 4b, the cell equipped with a Co_9_S_8_-PP separator exhibited ultra-stable overpotential cycling performance under a current density of 1 mA cm^−2^, and no significant fluctuation was observed within 2000 h. In contrast, the cell with the MOF-PP separator showed a higher overpotential, and the cell with a pure PP separator showed significant increases and fluctuations in overpotential after 500 h. The same trend could be observed when the current density increased to 3 mA cm^−2^ and 5 mA cm^−2^, respectively (Figure 4c,d). It is obvious that the separator modified by Co_9_S_8_ could effectively improve the cycling stability of the batteries. In contrast, the symmetric cells with PP and MOF-PP separators had higher voltage hysteresis and more pronounced increase and deviation in overpotential, probably attributed to the SEI layer’s repeated rupture and the formation of dead lithium during cycling. In addition, when the lithium deposition capacity increased to 2 mAh cm^−2^, the Li//Co_9_S_8_-PP-Co_9_S_8_//Li symmetric battery could achieve an ultra-stable voltage cycle of 1800 h at a current density of 2 mA cm^−2^ (Figure S10). If the lithium deposition capacity continues to increase to 3 mAh cm^−2^ with a current density of 1 mA cm^−2^, the symmetric cell can also maintain stability for 800 h (Figure S11).

Figure 4e illustrates the rate capability of the symmetric cells with different separators. The symmetrical cell with the Co_9_S_8_-PP separator exhibited outstanding rate performance at various current densities. The cell with Co_9_S_8_-PP separators had smaller overpotential and a gentler voltage change than the cell with PP and MOF-PP separators because the excellent morphology of the Co_9_S_8_-PP separator provides a faster transmission channel for Li^+^ and is conducive to charge transfer. In particular, the voltage plateau was able to return to its original level with a small voltage change as the current density changed from 8 mA cm^−2^ to 0.5 mA cm^−2^, indicating the good reversibility of the cell equipped with a Co_9_S_8_-PP separator at various current densities. These results demonstrate that the Co_9_S_8_-PP separator can effectively enhance the LMAs’ cyclic performance because of its unique nanowall structure and help eliminate inhomogeneous lithium deposition during cycling. In addition, the Co_9_S_8_ nanowall on the separator surface has a higher conductivity and can be used as an upper collector to accelerate electron transport, thus improving cycle stability and reversibility.

**Figure 5:**
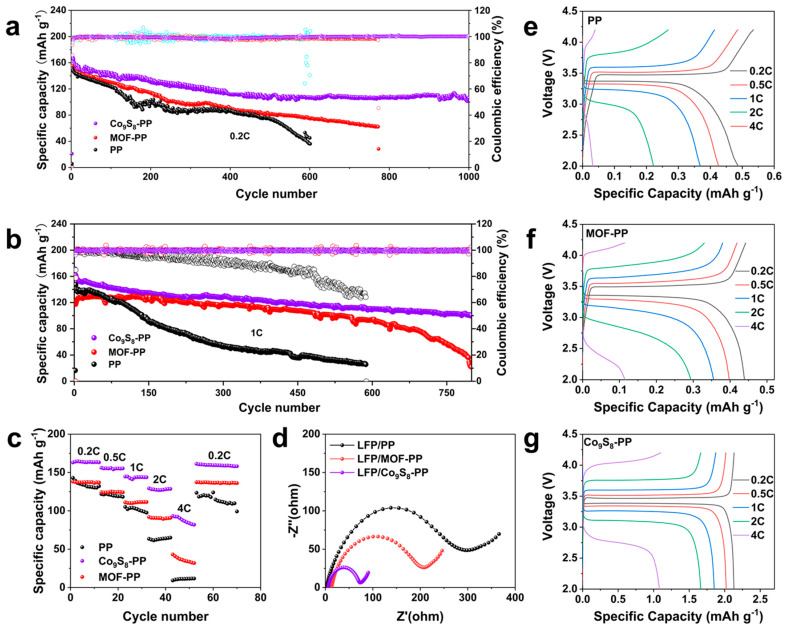
The cyclic performance of the Li//LFP full cells with different separators (**a**) at a 0.2 C rate and (**b**) at a 1C rate. (**c**) Rate characterization. (**d**) The Li/LFP full cells’ Nyquist plots. (**e**–**g**) The charge/discharge curves of PP, MOF-PP, and Co_9_S_8_-PP.

To evaluate the superiority of the Co_9_S_8_-PP separator in practical applications, the full cells using LiFePO_4_ (LFP) as a cathode were assembled to evaluate the electrochemical performance. As shown in Figure 5a, the full cell with a Co_9_S_8_-PP separator exhibited ultra-long cycle stability at 0.2 C with an initial capacity of 148 mAh g^−1^ and maintained 74% capacity after 1000 cycles. For commercial PP separators, the capacity decayed rapidly with the increase in the number of cycles, and only 26% of the capacity was retained after 600 cycles. When the rate increased to 0.5 C (Figure S12), the battery using Co_9_S_8_-PP was able to maintain 80% capacity over 800 cycles, while the battery with the PP separator could only keep 30% capacity after 450 cycles. Even at a high rate of 1 C, the Li//LFP full cells equipped with a Co_9_S_8_-PP separator still had a higher capacity retention and longer cycle stability than that of a PP battery (Figure 5b). In addition, Figure 5c shows that the cell using a Co_9_S_8_-modified separator also possessed a superior rate performance. When the current density gradually increased to 0.2, 0.5, 1, 2, and 4 C (Figure 5c), the discharge capacities of the Co_9_S_8_-PP cells were 164, 156, 143, 127, and 90 mAh g^−1^, while those of the PP cells were only 135, 121, 100, 62, and 11 mAh g^−1^. With the current density returning to 0.2 C, the Co_9_S_8_-PP cell was able to return to the initial stable high capacity, while the PP cell was unrecoverable, demonstrating the outstanding structural stability and electrochemical performance of the Co_9_S_8_-PP separator. The long-cycle performance of the full cells with different separators at various current densities proved that Co_9_S_8_ modification could significantly improve the cycle stability of PP separators. We speculate that the stable Co_9_S_8_ nanosheet array can effectively uniform the distribution of electric field during lithium stripping/plating, guide the uniform growth of lithium deposition, reduce the overpotential, and reduce the nucleation barrier.

Correspondingly, to evaluate the interfacial stability, the EIS of the full cells with LiFePO_4_ as a cathode was investigated. As shown in Figure 5d, the surface charge transfer resistance and interfacial resistance of the Co_9_S_8_-PP cell were significantly smaller than those of the PP cell, which demonstrated the outstanding interfacial stability of the full cell with the Co_9_S_8_-PP separator. We analyzed the first charge/discharge curves of the Co_9_S_8_-PP, MOF-PP, and bare PP separators. The voltage distribution of the LFP full cell at 0.2 C, 0.5 C, 1 C, 2 C, and 4 C is shown in Figure 5e–g. In contrast, the LFP/Co_9_S_8_-PP cells possessed a lower voltage plateau than the LFP/bare PP cells. The Co_9_S_8_ arrays are more likely to lower the polarization of the LMAs during charging and discharging, resulting in a low voltage plateau, proving faster charge transfer kinetics at the interface layer between the Co_9_S_8_ layer and the electrolyte. Notably, the Co_9_S_8_-PP separator-based full cells showed higher discharge capacity than bare PP separator full cells, especially at a high current density. Bare lithium foil does not allow for uniform lithium stripping, and most of the activated lithium forms “dead lithium” between the PP separator and the lithium foil, resulting in this significant difference. Conversely, a high-conductivity Co_9_S_8_ layer allows the lithium to be uniformly stripped/plated without the formation of “dead lithium”. Consequently, advanced modified separators, such as Co_9_S_8_-PP separators, have higher theoretical capacities in full cells, which paves a scalable route to prepare lithiophilic diaphragms for practical applications of lithium metal anodes in high-performance lithium batteries.

**Figure 6:**
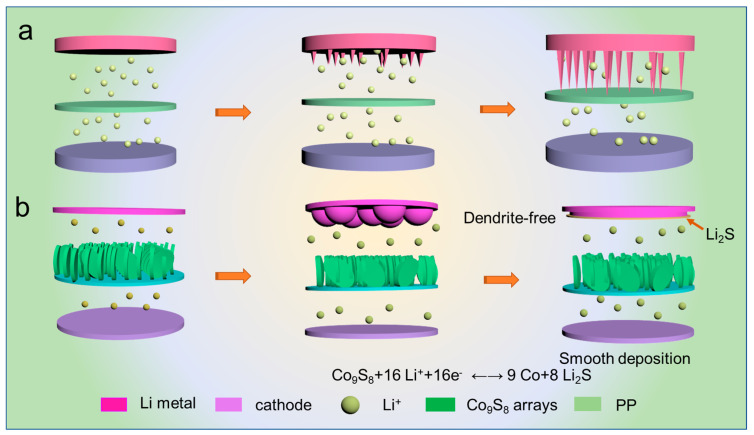
A schematic diagram of the lithium electrodeposition behavior on the LMAs of the full cells with (**a**) PP, and (**b**) Co_9_S_8_-PP separators.

As described in Figure 6, we schematically illustrate the lithium deposition behavior of the commercial separator and Co_9_S_8_-PP separator in Li//LFP full cells. Because of the high reactivity of lithium metal, a series of side reactions occur when lithium metal is exposed to the carbonate-based electrolyte in conventional LMBs, which can lead to the formation of small, needle-like lithium particles on the anode surface during charging (Figure 6a). As the cycle time increases, these needle-like lithium particles grow into dendritic lithium dendrites and lead to the electrolyte being exposed to more lithium, exacerbating side reactions and generating large amounts of “dead lithium” which reduce the efficiency of the cell. The large number of formed lithium dendrites can easily pierce the original SEI layer and eventually leads to cell failure. Notably, after growing a layer of Co_9_S_8_ arrays in situ on the PP separator, the performance of the cells improved significantly. For the full cells equipped with Co_9_S_8_-modified separators (Figure 6b), the excellent morphology of the Co_9_S_8_ array provides a channel for fast Li^+^ transport, while the high electrolyte absorption efficiency implies that the modified separator can store more electrolyte to ensure the continuation of the reaction. More importantly, the Co_9_S_8_ will form Li_2_S with Li^+^ during the charge and discharge process, which will enhance the ionic conductivity of the Co_9_S_8_-PP separator, significantly accelerate the transport of Li^+^, and help homogenize Li^+^ flux. These can suppress the “tip effect” and promote smooth and uniform lithium deposition. Meanwhile, the generated Li_2_S can contribute to stabilizing the SEI, and the stable SEI can avoid the formation of lithium dendrites and reduce the formation of “dead lithium”, which significantly improves the capacity efficiency of the full cells.

## 4. Conclusions

In summary, in order to solve a series of hazardous problems caused by uncontrollable dendrite growth in lithium metal anodes, we provide a strategy to suppress dendrite growth by modifying PP separators with transition metal sulfides. The Co_9_S_8_ nanowall array has a good morphology with a uniform distribution, which provides a basis for uniform Li^+^ transport. The Co_9_S_8_-modified layer effectively stabilizes lithium electrodeposition, while the high electrolyte absorption efficiency improves lithium utilization. This advanced modified separator helps to improve the Coulomb efficiency, reduces the battery polarization, and effectively improves the reversibility and cycling stability of the lithium anode after long-term cycling. Additionally, the prepared modified separator can significantly enhance the rate performance and long-cycle stability of the full cell when coupled with a LiFePO_4_ cathode. The preparation of this modified separator is suitable for large-scale fabrication, implying that the present work provides new ideas to promote the practical application of lithium metal anodes.

## Data Availability

The original contributions presented in the study are included in the article/Supplementary Material, further inquiries can be directed to the corresponding author/s.

## Supplementary Materials

The following supporting information can be downloaded at: https://www.mdpi.com/article/10.3390/polym16131924/s1, Figure S1: (a) Optical photo of polypropylene (PP) separator. ((b) and (c)) SEM image of PP separator; Figure S2: Optical photos of as-prepared MOF-PP separator: (a) Front side, (b) back side, and (c) folded. (d) mass production; Figure S3: Element mapping images and atomic mass fractions corresponding to carbon and cobalt; Figure S4: XRD patterns of PP and Co_9_S_8_-PP separators; Figure S5: Element mapping images and atomic mass fractions corresponding to carbon, cobalt and sulfur; Figure S6: Optical photos of as-prepared Co_9_S_8_-PP separator: (a)Front side, (b) folded; Figure S7: The thickness of (a) PP separator. (b) MOF-PP. (c) Co_9_S_8_-PP; Figure S8: XPS spectra of Co_9_S_8_; Figure S9: The electrolyte uptake results of different separators. Here, *W_i_* is the mass of the dry sample, *W_f_* is the weight of the electrolyte-soaked sample, and *E_u_*, the electrolyte uptake ratio, was calculated according to the inset equation; Figure S10: Electrochemical long-term cycling with current density of 2 mA cm^−2^ and capacity of 2 mAh cm^−2^; Figure S11: Electrochemical performance with current density of 1 mA cm^−2^ and capacity of 3 mAh cm^−2^; Figure S12: Electrochemical long-cycle performance of Li/LFP full cell at 0.5 C rate.
