# Supplementary material for: Metal–Organic Framework-Derived Co9S8 Nanowall Array Embellished Polypropylene Separator for Dendrite-Free Lithium Metal Anodes
Source: Polymers (Basel). 2024 Jul 5;16(13):1924. doi: 10.3390/polym16131924 (PMC11244197; doi:10.3390/polym16131924)
Supplement: Supplementary file 1 [file polymers-16-01924-s001.zip › polymers-3036510-supplementary.pdf]

Supporting Information

MOF-derived Co_9S_8 Nanowall Array Embellished Polypropylene Separator for Dendrite-free Lithium Metal Anodes

Deshi Feng[a§], Ruiling Zheng[b§], Li Qiao[a,c§], Shiteng Li[d], Fengzhao Xu[a], Chuangen Ye[a], Jing Zhang[a]*, Yong Li[a]*

[a] Advanced Materials Institute, School of Materials Science and Technology, Qilu University of Technology (Shandong Academy of Sciences), Jinan, 250014, China.
[b] Shandong Communication & Media College, Jinan, 250200, China.
[c] SVOLT Energy Technology Co., Ltd, Changzhou, 213299, China.
[d.] College of Materials and Chemical Engineering, Heilongjiang Institute of Technology, Harbin, 150006, China.

[§] These authors contributed equally to this work.
* Corresponding author
Email: yongli@sdas.org, jzhang@qlu.edu.cn

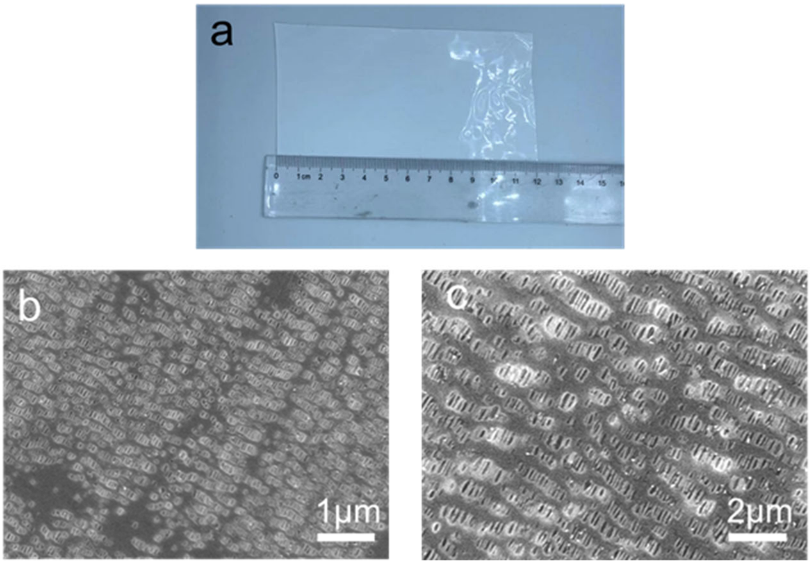

Figure S1 (a) Optical photo of polypropylene (PP) separator. (b and c) SEM image of PP separator.

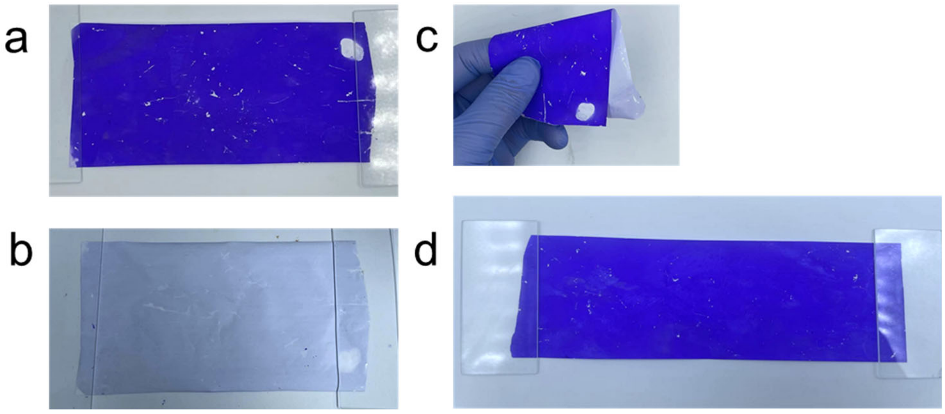

Figure S2 Optical photos of as-prepared MOF-PP separator: (a) Front side, (b) back side, and (c) folded. (d) mass production.

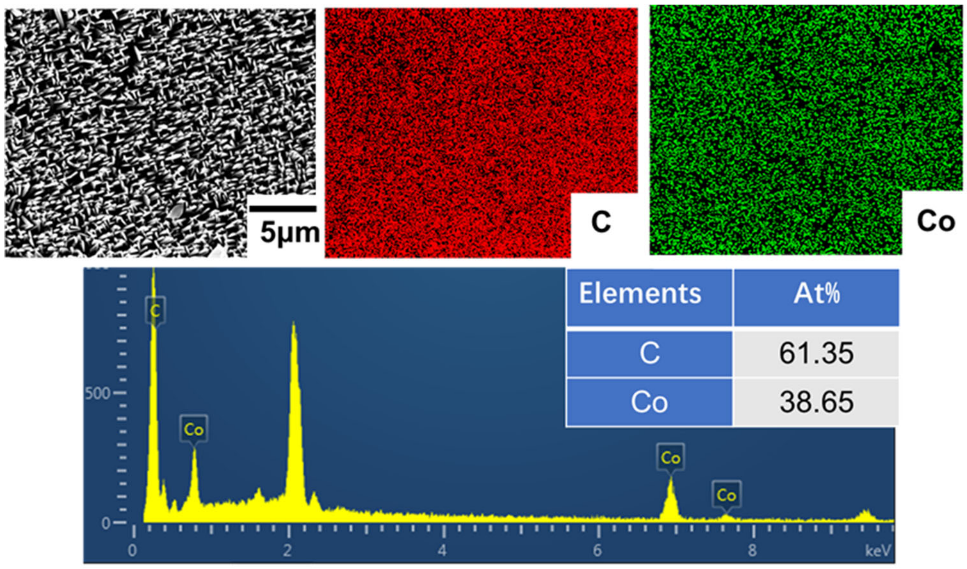

Figure S3 Element mapping images and atomic mass fractions corresponding to carbon and cobalt.

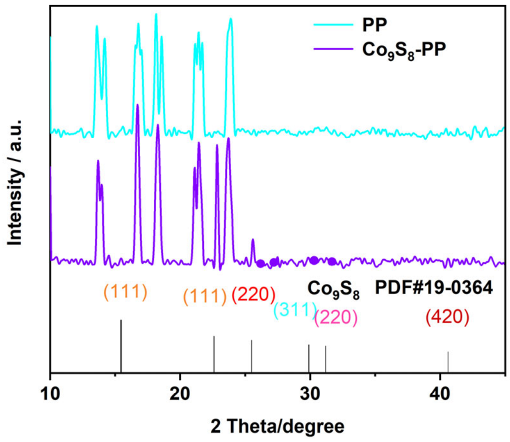

Figure S4 XRD patterns of PP and Co_9S_8-PP separators.

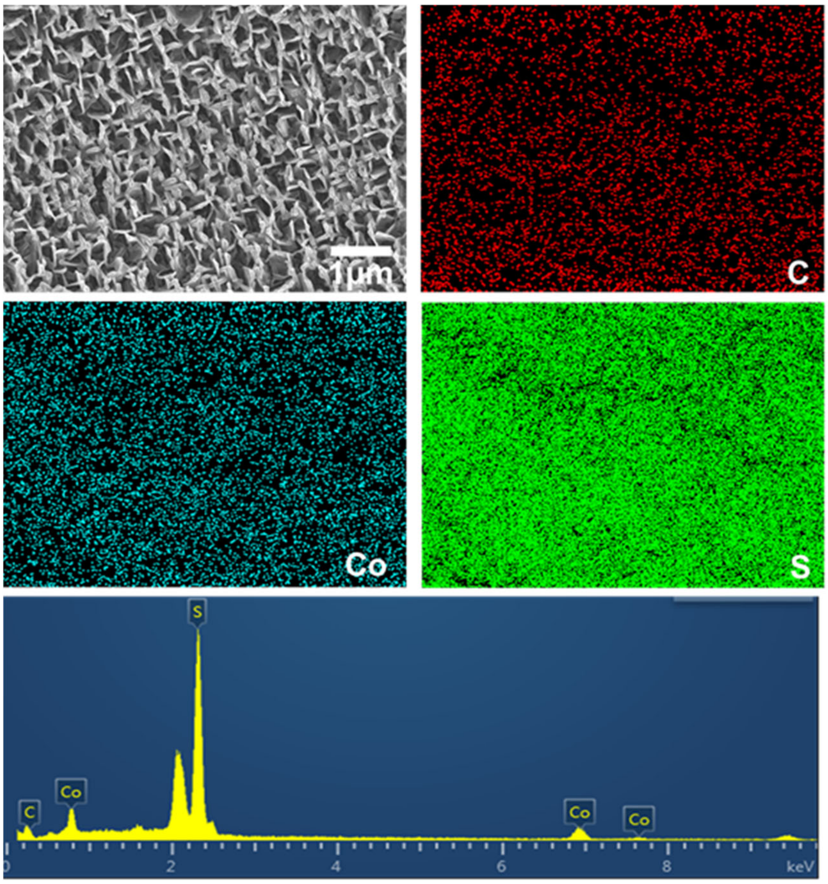

Figure S5 Element mapping images and atomic mass fractions corresponding to carbon, cobalt and sulfur.

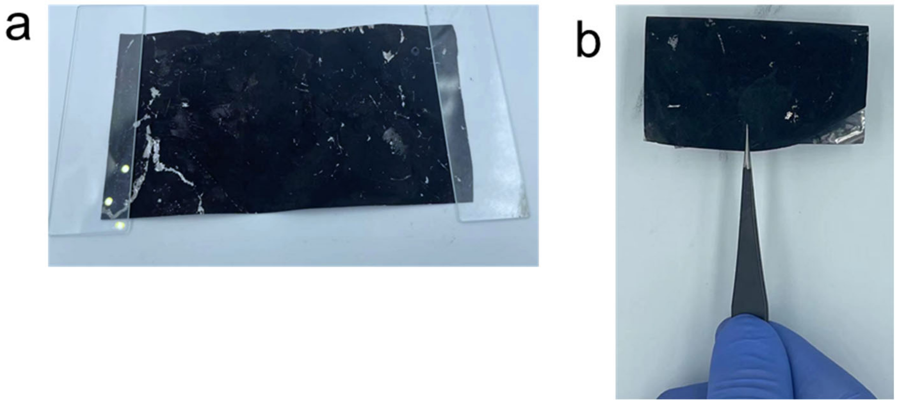

Figure S6 Optical photos of as-prepared Co_9S_8-PP separator: (a)Front side, (b) folded.

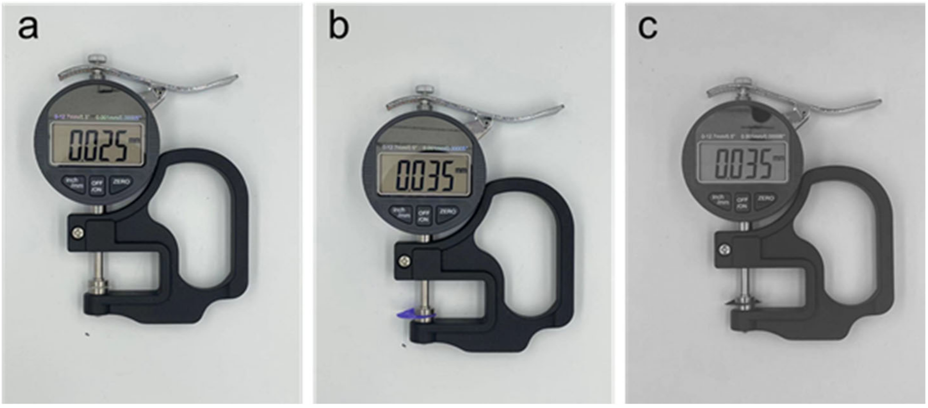

Figure S7 The thickness of (a) PP separator. (b) MOF-PP. (c) Co_9S_8-PP.

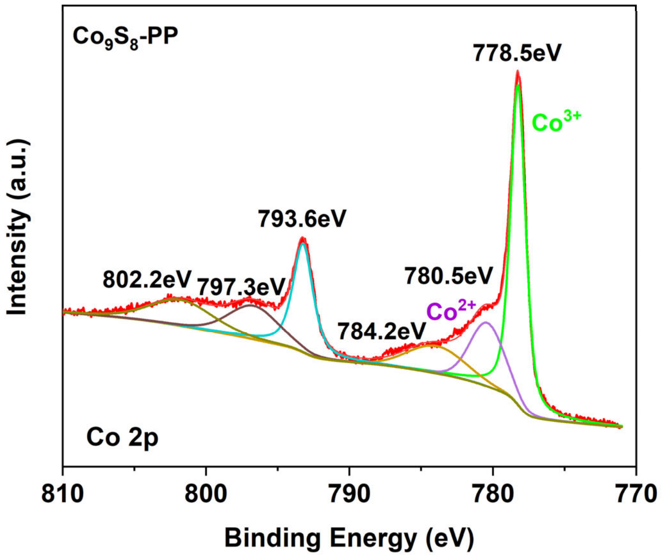

Figure S8 XPS spectra of Co_9S_8.

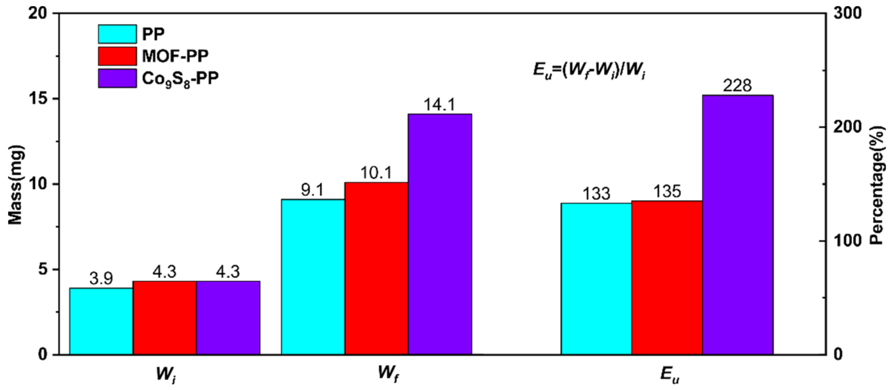

Figure S9 The electrolyte uptake results of different separators. Here, W_i is the mass of the dry sample, W_f is the weight of the electrolyte-soaked sample, and E_u, the electrolyte uptake ratio, was calculated according to the inset equation.

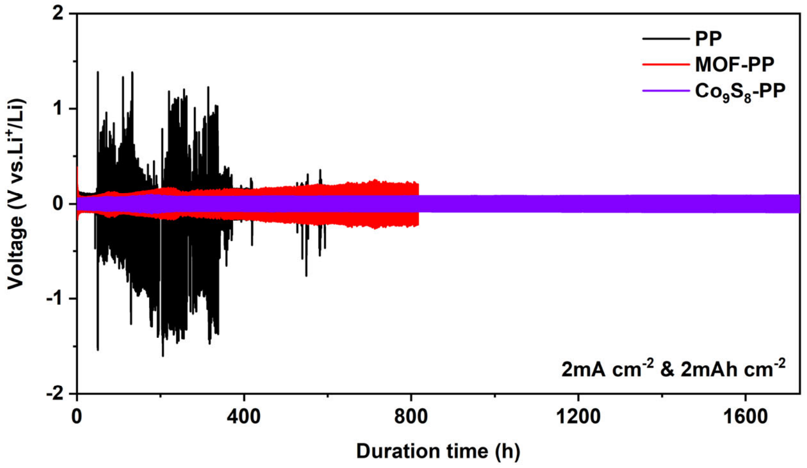

Figure S10 Electrochemical long-term cycling with current density of 2mA cm^{-2} and capacity of 2mAh cm^{-2}.

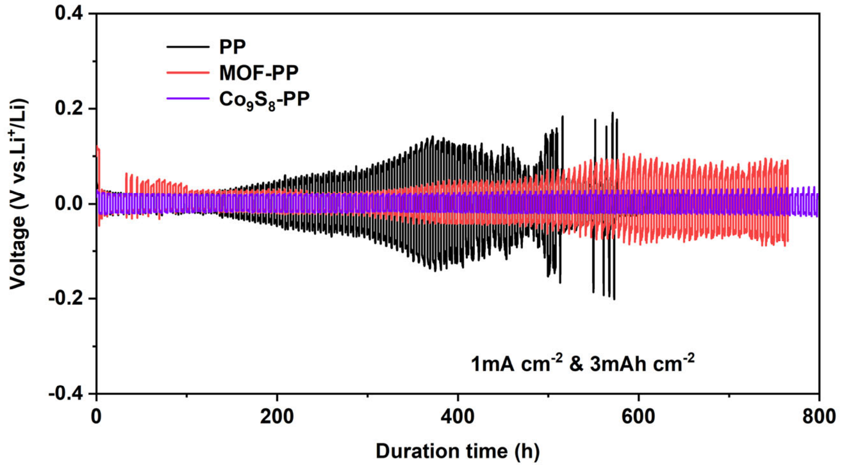

Figure S11 Electrochemical performance with current density of 1mA cm^{-2} and capacity of 3mAh cm^{-2}.

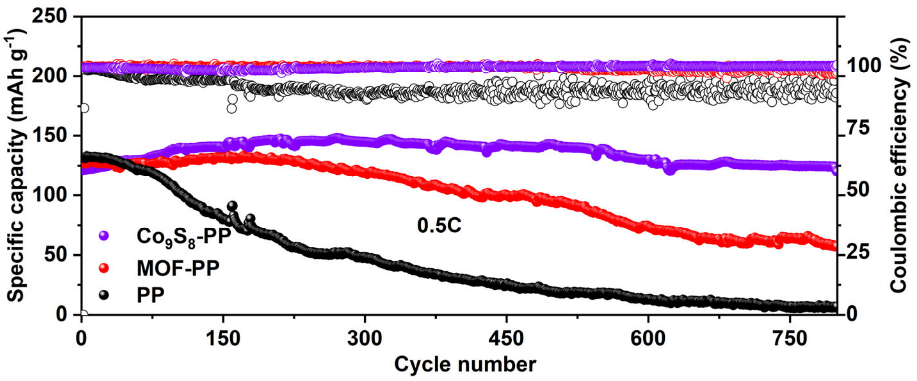

Figure S12 Electrochemical long-cycle performance of Li/LFP full cell at 0.5 C rate.
